# Physiological characterization and transcriptome analysis of *Pichia pastoris* reveals its response to lignocellulose-derived inhibitors

**DOI:** 10.1186/s13568-020-01170-9

**Published:** 2021-01-03

**Authors:** Barbara G. Paes, Andrei Stecca Steindorff, Eduardo F. Formighieri, Ildinete Silva Pereira, João Ricardo M. Almeida

**Affiliations:** 1 Laboratory of Genetics and Biotechnology, Embrapa Agroenergia, Parque Estação Biológica, PqEB – W3 Norte Final s/no, Brasília, DF 70.770-901 Brazil; 2 Graduate Program of Molecular Biology, Department of Cell Biology, Institute of Biology, University of Brasilia, Brasília, Brazil; 3 Graduate Program of Microbial Biology, Department of Cell Biology, Institute of Biology, University of Brasilia, Brasília, Brazil

**Keywords:** *Komagataella phaffii*, *Pichia pastoris*, Inhibitors, Lignocellulosic hydrolysate, Acetic acid, Furaldehydes

## Abstract

The negative effects of lignocellulose-derived inhibitors such as acetic acid and furaldehydes on microbial metabolism constitute a significant drawback to the usage of biomass feedstocks for the production of fuels and chemicals. The yeast *Pichia pastoris* has shown a great biotechnological potential for producing heterologous proteins and renewable chemicals. Despite its relevance, the performance of *P. pastoris* in presence of lignocellulose-derived inhibitors remains unclear. In this work, our results show for the first time the dose-dependent response of *P. pastoris* to acetic acid, furaldehydes (HMF and furfural), and sugarcane biomass hydrolysate, both at physiological and transcriptional levels. The yeast was able to grow in synthetic media with up to 6 g.L^−1^ acetic acid, 1.75 g.L^−1^ furaldehydes or hydrolysate diluted to 10% (v/v). However, its metabolism was completely hindered in presence of hydrolysate diluted to 30% (v/v). Additionally, the yeast was capable to co-consume acetic acid and glucose. At the transcriptional level, *P. pastoris* response to lignocellulose-derived inhibitors relays on the up-regulation of genes related to transmembrane transport, oxidoreductase activities, RNA processing, and the repression of pathways related to biosynthetic processes and central carbon metabolism. These results demonstrate a polygenetic response that involves detoxification activities, and maintenance of energy and cellular homeostasis. In this context, *ALD4, OYE3, QOR2, NTL100, YCT1,* and *PPR1* were identified as target genes to improve *P. pastoris* tolerance. Altogether, this work provides valuable insights into the *P. pastoris* stress tolerance, which can be useful to expand its use in different bioprocesses.

## Introduction

Lignocellulosic biomass is an abundant raw material that can be converted by physicochemical and microbial processes into different products, such as biofuels, building-block chemicals, and high added-value chemicals (Anwar et al. 2014; Paes and Almeida 2014). Before microbial fermentation, the biomass needs to undergo pretreatment and hydrolysis to release the monosaccharides present in the biomass. During pretreatment, compounds that inhibit microbial metabolism are also released or formed during dehydration of pentoses and hexoses, hemicellulose deacetylation, or lignin breakdown (Almeida et al. 2007; Jönsson and Martín 2016). These inhibitors can be classified into three main groups: furaldehydes, such as 2-furaldeyde (furfural) and 5-hydroxymethyl-2-furaldehyde (HMF), weak acids (acetic acid, formic acid, and levulinic acid), and phenolic compounds (vanillin, syringaldehyde, coniferyl aldehyde, and othersAlmeida et al. 2007; Hasunuma and Kondo 2012). The pretreatment and hydrolysis procresses, as well as biomass source influence the formation and concentrations of the aforementioned compounds in lignocellulosic hydrolysates (Almeida et al. 2011; Hasunuma and Kondo 2012; Jönsson and Martín 2016).

Effects of lignocellulose-derived inhibitors on yeast physiology and resistance mechanisms have been extensively investigated for *Saccharomyces cerevisiae* (Rumbold et al. 2009; Almeida et al. 2011; Zha et al. 2013; Yang et al. 2018) and to a minor extent for other yeasts, like *Zygosaccharomyces* (Martín and Jönsson 2003)*, Spathaspora passalidarum* (Hou and Yao 2012), *Candida* spp (Cottier et al. 2015; Moreno et al. 2019) and others (Delgenes et al. 1996; Zha et al. 2013; Yamakawa et al. 2020). Inhibitory effects and mechanisms vary depending on the chemical structure of the specific inhibitor and its concentration. Generally, they are cytotoxic and hinder microbial growth, reduce cell vitality and fermentation efficiency. Their main mechanisms of action involve inhibition of essential enzymes related to cell metabolism, DNA replication, RNA, and protein synthesis and redox imbalance, and damaging cellular membranes (Modig et al. 2002, 2008; Liu et al. 2004; Almeida et al. 2007; Skerker et al. 2013; Sitepu et al. 2015).

The *S. cerevisiae* response to inhibitors is complex and involves a polygenetic modulation of various metabolic pathways, such as carbon, lipid, amino acid metabolism, and regulatory pathways, among others. The differential gene expression redirects the yeast’s metabolism to allow repair of damages caused by the inhibitors and increase the innate detoxification activities (Petersson et al. 2006; Almeida et al. 2009; Mira et al. 2010; Adeboye et al. 2015; Brandt et al. 2019). The understanding of such complex mechanisms in yeasts of industrial interest is, therefore, crucial. Among those, methylotrophic yeasts, such as *Ogatae polymorpha* and *Pichia pastoris*, can be highlighted given their role in the production of fuels and chemicals (Radecka et al. 2015). On this matter, recent observations pointed to *O. polymorpha* tolerance to wheat straw hydrolysate when containing different concentrations of acetic acid, formic acid, furaldehydes, and phenolic compounds. The results demonstrated that the sugar uptake by the yeast was reduced in the presence of inhibitors. The yeast was still able to consume some xylose and produce xylitol in presence of 12.24 g.L^−1^ of acetic acid and 4.17 g.L^−1^ of total phenolics (Yamakawa et al. 2020). However, the methylotrophic yeast response mechanisms to the inhibitors were not previously reported.

As previously mentioned, the yeast *Komagataella phaffii*, previously known and here referred to as *Pichia pastoris* (Gasser & Mattanovich, 2018) is a methylotrophic yeast extensively used in the production of heterologous proteins and metabolites both in industry and academia (Zahrl et al. 2017). To this date, more than five thousand different proteins have been heterologously expressed in this yeast (Schwarzhans et al. 2017). The biotechnological potential of *P. pastoris* has been amplified by its use in metabolic engineering programs (Nocon et al. 2014; Peña et al. 2018), and the production of many other compounds besides proteins have been considered, including alcohols, acids, vitamins, and others (Siripong et al. 2018)(Vogl et al. 2013; Almeida et al. 2018; Gasser and Mattanovich 2018; Melo et al. 2018). The increasing interest in *P. pastoris* has led to the construction of recombinant strains capable of metabolizing carbon sources derived from lignocellulose, including cellulose (Kickenweiz et al. 2018), glucose (Siripong et al. 2018) and xylose (Li et al. 2015; Almeida et al. 2018). Acetic acid has also been studied as an alternative carbon source for this yeast (Xie et al. 2005; Xu et al. 2019).

Here we unveil the potential of *P. pastoris* for the conversion of sugars present in lignocellulosic hydrolysates. More specifically, we evaluate the yeast’s physiological response to acetic acid, furaldehydes, and sugarcane biomass hydrolysate. RNA-seq based transcriptome analysis was employed to investigate the global response of *P. pastoris* in the presence of different concentrations of those compounds. Lastly, the physiological and transcriptional dose-dependent response of *P. pastoris* to the inhibitors are presented and discussed.

## Methods

### Strain and media

The yeast *P. pastoris* X33 was used in this work (Invitrogen, USA). Stock cultures of yeast grown in YPD medium (1% w/v yeast extract, 2% w/v peptone, 2% w/v glucose) were preserved in 30% glycerol and maintained at − 80 °C.

In order to evaluate the effect of inhibitors on yeast metabolism, the medium employed was composed of (w/v): YNB (yeast nitrogen base)(Sigma Aldrich Y0626) without amino acids (0.68% YNB, 2% ammonium sulfate), 2% glucose, 4% xylose, buffered to pH 5.5 with phthalate buffer (5.1% potassium hydrogen phthalate with 1,1% potassium hydroxide w/v). For each culture condition, inhibitory compounds were added to the media in the following concentrations 2 and 6 g.L^−1^ of acetic acid; a mixture of 0.9 g.L^−1^ furfural and 0.15 g.L^−1^ HMF (FH 0.9/0.15 g.L^−1^) and 1.5 g.L^−1^ furfural and 0.25 g.L^−1^ HMF (FH 1.5/0.25 g.L^−1^) for furaldehydes; and sugarcane bagasse hydrolysate diluted to 10% and 30% of the initial concentration. The sugarcane bagasse hydrolysate was obtained by steam explosion of sugarcane bagasse, than for the breakdown of the oligomers in the hemicellulose-rich fraction, the liquid fraction of the steam explosion was subjected to hydrolysis with 0.5% H_2_SO_4_ (w/w) at 130 °C for 100 min (Morais Junior et al. 2019). The final composition of the sugarcane bagasse hydrolysate was: 5.4 g.L^−1^ glucose, 90.3 g.L^−1^ xylose, 19.4 g.L^−1^ acetic acid, 2.9 g.L^−1^ furfural, 0.55 g.L^−1^ HMF. In the media containing diluted hydrolysate, the amount of glucose and xylose present in the hydrolysate was accounted to keep the final glucose and xylose concentration at 2% and 4%, respectively.

### Culture conditions

Cells plated in YPD medium were initially inoculated in 5 mL YPD and grown overnight (28 °C, 200 rpm on a rotary shaker). Then cells were transferred to 200 mL YPD in a 1 L shake flasks and grown overnight at the same conditions. The culture was washed twice with distilled water and diluted down to an initial optical density (OD) at 600 nm of 5 in 50 mL of medium in 250 mL shake flasks. The culture was incubated for 30 h at 28 °C and 200 rpm. Samples for transcriptome were withdrawn after 4 h of incubation and samples for metabolite analysis were withdrawn regularly. All experiments were carried out in biological triplicate.

### RNA extraction and quality analysis

RNA was extracted using TRIZOL (Thermo Fisher Scientific, USA) reagent following the manufacturer´s protocol with few modifications. A culture of 5 mL was harvested for 1 min at 14.000 × *g* at 4 °C. The supernatant was discarded and 1 mL of TRIZOL was added to the pellet. Cells were transferred to a 2 mL microtube containing approximately 200 μL of sterile 0.02 mm glass beads and then disrupted by four cycles of 1 min at Mini-Beadbeater-96 (Biospec Products, USA), resting the tube on ice between cycles. Finally, RNA extraction followed manufacturer’s instructions by performing chloroform and ethanol washings. The RNA integrity was evaluated via Agilent Bioanalyzer 2100 system (Agilent Technologies, USA), Nanodrop 1000 Spectrophotometer (Thermo Fisher Scientific, USA) and in 1% agarose gels.

### RNA sequencing and data analysis

RNA-seq was performed by Centro de Genômica, the University of São Paulo on Illumina HiSeq 2500 system v4 using HiSeq SBS Kit v4, and 100 bp (2x) paired-end reads. Libraries for RNA-Seq were prepared with TruSeq Stranded mRNA Sample Prep LT Protocol (Illumina, USA) from RNA extractions of 21 independent samples. FastQC software was used to evaluate base quality distributions based on phred value (Andrews et al. 2012). Raw reads were processed with Trimmomatic software (Bolger et al. 2014), and once again analyzed for the quality of clean sequences on FastQC. Sequences were aligned using STAR (Dobin et al. 2013). HTSeq-count version 0.9.1 tool (Anders et al. 2015) was used for counting the number of aligned sequences for each sample in each gene and estimate gene expression. Differentially expressed genes were detected by entering the count data into the R program (R Core Team 2020) and using the DESeq2 package (Love et al. 2014).

Genes were considered significantly differentially expressed with an adjusted *P*-values limit < 0.05 both for increasing and decreasing expression. The differentially expressed genes (DEG) overlap between conditions was assessed using Venn diagrams built with the Venn online platform (http://bioinformatics.psb.ugent.be/webtools/Venn/). The list of DEGs in the DESeq2 package was used for functional analysis to identify which genes and metabolic pathways are being activated or repressed in response to acetic acid, furaldehydes and hydrolyzed. For this, the induced and the repressed genes were separated into different files. The individual lists were subjected to functional enrichment analysis using Fisher's Exact Test with a false discovery rate (FDR) < 0.05 in the GO_MWU tool (https://github.com/z0on/GO_MWU). This analysis assesses the significance of the representativeness of the GO (Gene Ontology) categories among DEG.

For the heatmap, the expression values of 630 genes differentially expressed in all conditions (adjusted *P*-value ≤ 0.05) were hierarchically clustered using MeV 4.9.0 program (http://mev.tm4.org) with Pearson correlation metric and average linkage clustering. A distance threshold of 0.75 was used to split the gene tree into 7 clusters. Gene ontology annotation from each cluster was used as input to REVIGO (Supek et al. 2011) analysis to reduce redundancy and build the network. We used GO terms database from *S. cerevisiae* and SimRel as the semantic similarity measure.

The transcriptome datasets generated during the current study are available in the NCBI with the accession number PRJNA666642.

### Quantification of metabolites

Carbon sources (xylose and glucose) and extracellular metabolites xylitol, glycerol, acetate, HMF, and furfural concentrations were determined by High-Performance Liquid Chromatography (HPLC) (Veras et al. 2017) in samples withdrawn on different time points. Samples were centrifuged, and the supernatant was analyzed by HPLC (Acquity UPLC H Class, Waters, USA) equipped with a refractive index and a PDA detector. Metabolites were separated on an HPX-87 H column (Bio-Rad Laboratories, USA), using a 5 mM sulfuric acid mobile phase at a flow rate of 0.6 mL/min and temperature of 45 °C. Biomass was measured through OD_600_ using a spectrophotometer (SpectraMax M3, Molecular Devices, USA).

## Results

### Fermentative performance of *P. pastoris* in presence of lignocellulose-derived inhibitors

To gain insight of the physiological response of *P. pastoris* to lignocellulose-derived inhibitors, the yeast was cultivated in the presence of acetic acid, furaldehydes, and sugarcane bagasse hydrolysate. As the yeast growth was completely abolished in hydrolysate concentrations above 30% (data not shown), the sugarcane biomass hydrolysate employed in this study was diluted in defined medium to 10% and 30% concentrations. The concentrations of acetic acid (2 g.L^−1^ and 6 g.L^−1^) and furaldehydes (FH 0.9/0.15 g.L^−1^ and FH 1.5/0.25 g.L^−1^) were similar to the ones found in the hydrolysate 10% and 30%. Similar concentrations were always observed in the range of inhibitors found in lignocellulosic hydrolysates from different sources after physicochemical pretreatment (Kim 2018).

**Fig. 1:**
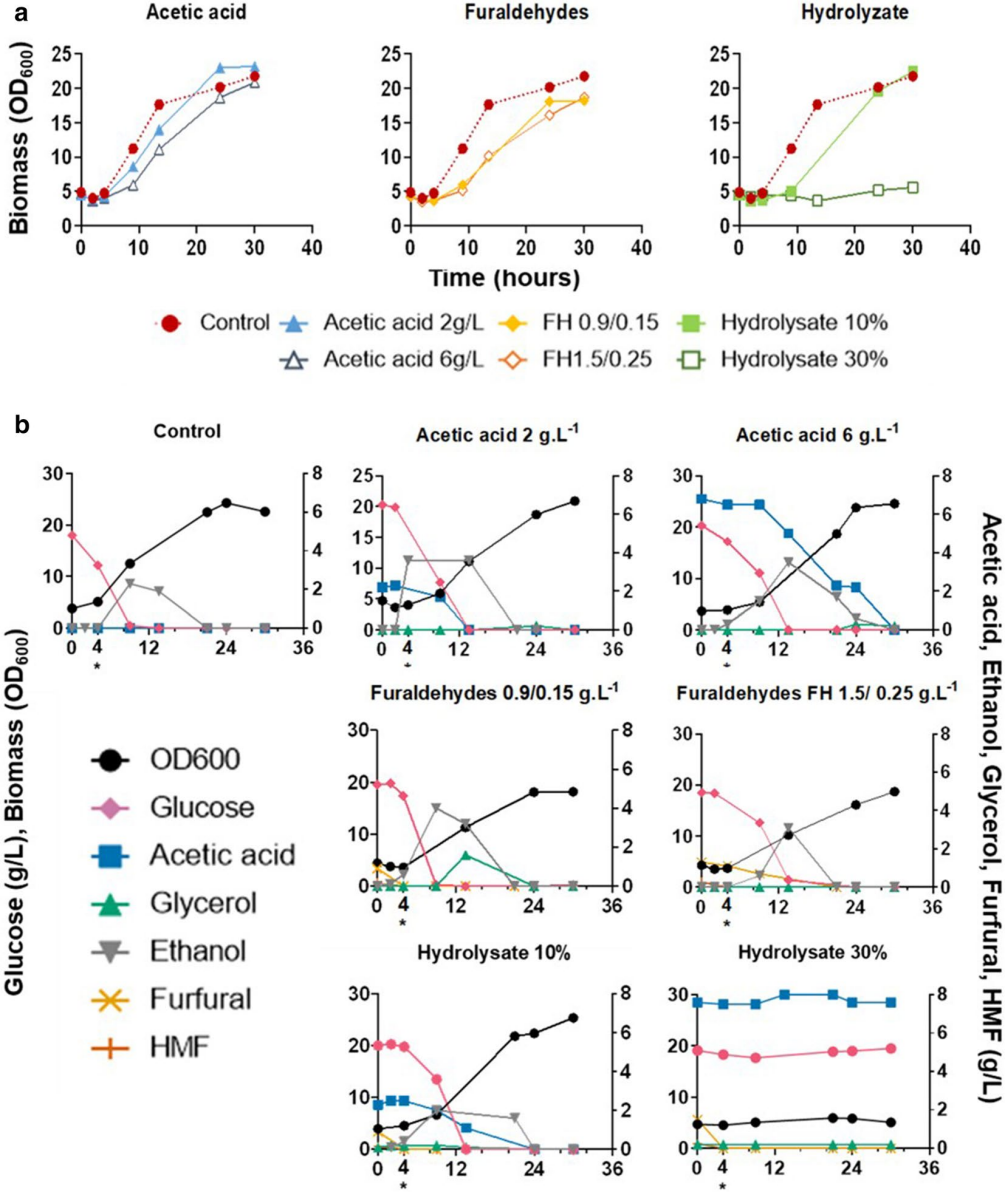
*P. pastoris* growth profile in the presence of different inhibitors. **a** growth curves of: acetic acid (2 g.L^−1^—closed triangle, and 6 g.L^−1^ open triangle), furaldehydes (0.15 g.L^−1^ HMF, 0.9 g.L^−1^ furfural—closed diamond and 0.25 g.L^−1^ HMF, 1.5 g.L^−1^ furfural—open diamond), and hydrolysate (10%—closed square, and hydrolysate 30% open square) against control (closed circle with dotted lines). **b** substrate consumption and product formation in different conditions: control, acetic acid 2 g.L^−1^, acetic acid 6 g.L^−1^, FH 0.9/ 0.15 g.L^−1^, FH 1.5/ 0.25 g.L^−1^, hydrolysate 10%, hydrolysate 30%. Biomass (OD600, black circle), glucose (pink diamond), acetic acid (blue square), glycerol (green triangle), ethanol (gray upside-down triangle), furfural (yellow star), HMF (orange cross). Timepoint 4* highlights the timepoint where samples were taken. Xylose concentration was constant through the cultivation. The experiments were performed in triplicate and the figure represents the profile of one replicate

The growth profile of *P. pastoris* varied drastically according to the conditions evaluated (Fig. 1a). In the absence of inhibitors, the lag phase of growth ended after 4 h, the yeast consumed 97% of the available glucose after 9 h of cultivation reaching OD_600_ around 18 (Fig. 1a). Acetic acid did not extend the lag phase, but it reduced the yeast growth and sugar consumption rate in the first hours of cultivation. Indeed, the yeast consumed 81% and 60.6% of available glucose in the presence of 2 g.L^−1^ and 6 g.L^−1^ of acetic acid, respectively, compared to the control (Fig. 1b). However, the final yeast growth in 2 g.L^−1^ of acetic acid was slightly higher than in control (OD = 22,8 ± 1 compared to 25,3 ± 2). In that case, the yeast was able to fully consume all the acetic acid present in the medium.

In the presence of furaldehydes, the yeast showed an extended lag phase, with its final growth reduced in in approximately 14% when compared to the control condition (Fig. 1a). The prolonged lag phase correlated with reduced sugar consumption in both concentrations of furaldehydes (Fig. 1b). Indeed, the yeast consumed only 61% and 47% of the available glucose in the first 9 h of fermentation, respectively.

The hydrolysate had the most negative impact on the yeast metabolism (Fig. 1a). Even at the lowest concentration of hydrolysate, the yeast showed an extended lag phase and reduced sugar consumption rate when compared to the control. Upon inhibition *P. pastoris* consumed only 57.1% of glucose after 9 h when cultivated in the medium with 10% hydrolysate compared to 97% in the medium without inhibitors (Fig. 1b). However, after 30 h of cultivation, the yeast was able to reach similar final growth in the presence of the hydrolysate 10% and in the control media. It was also able to consume the acetic acid present in the hydrolysate. Hydrolysate 30% completely impaired the yeast metabolism (Fig. 1b), and no growth was detected even after 72 h of incubation (data not shown). Of note, in the evaluated conditions, *Pichia* was not able to consume xylose.

### Transcriptional response of *P. pastoris* towards lignocellulose-derived inhibitors

**Fig. 2:**
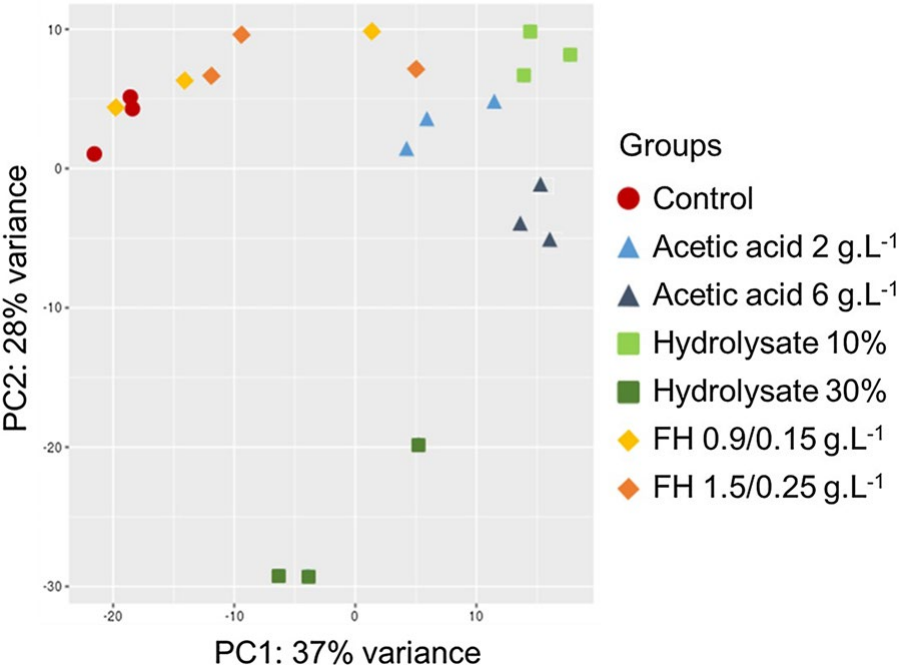
Principal component analysis (PCA) of RNA-seq data of *P. pastoris* cultivated in different inhibitors. Dots represent samples and are colored according to the different conditions investigated: red circle: Control—minimal medium without inhibitor, light blue triangle: acetic acid 2 g.L^−1^, dark blue triangle: acetic acid 6 g.L^−1^, light green square: hydrolysate 10%, dark green square: hydrolysate 30%, yellow diamond: FH 0.9/ 0.15 g.L^−1^, and orange diamond: FH 1.5/ 0.25 g.L^−1^

A genome-wide RNA-seq transcriptional profiling was used to understand the overall cellular response of *P. pastoris* toward lignocellulose-derived inhibitors*.* For this, the yeast was cultivated in YNB medium supplemented or not with two different concentrations of acetic acid, furaldehydes (HMF and furfural) or sugarcane bagasse hydrolysate*.* Glucose and xylose concentrations were normalized to 2% and 4%, respectively, in all cultivation conditions (Fig. 1). To identify differentially expressed genes (DEGs), the experimental data from cultivations in presence of inhibitors were normalized to the control condition (no inhibitor). A total of 429,738 sequence reads were obtained after quality trimming. Samples were aligned to the *K. phaffii* str. WT (GenBank accession no. GCA_001708085) reference genome. Principal component analysis based on expression patterns showed a good reproducibility of the biological replicates and distinct isolation of hydrolysate 30% replicates from the other conditions (Fig. 2).

**Fig. 3:**
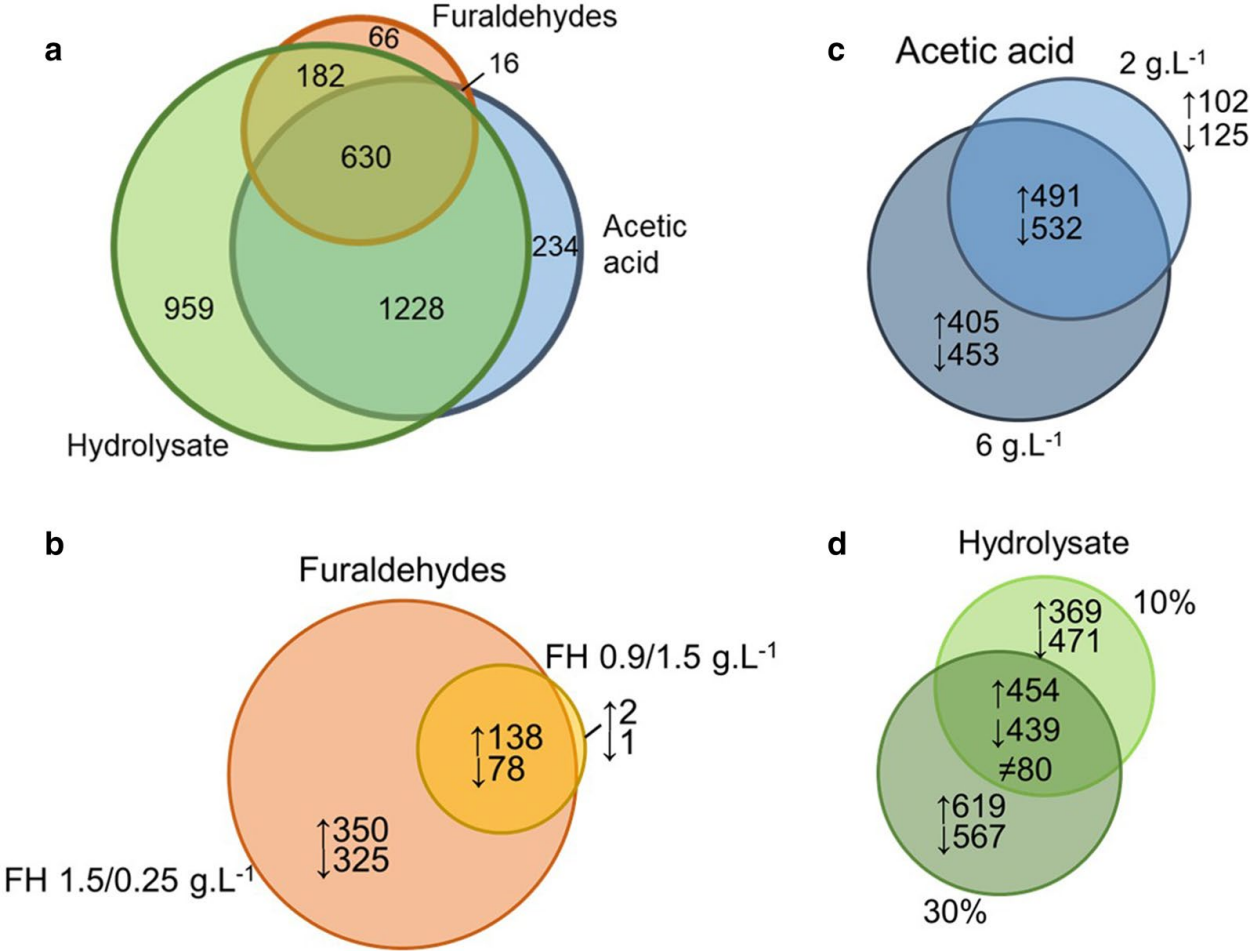
Venn diagrams representing *P. pastoris* differentially expressed genes (DEGs) in response to lignocellulose-derived inhibitors. **a** three inhibitors, no concentration differentiation; **b** acetic acid 2 g.L^−1^ and 6 g.L^−1^; **c** furaldehydes FH 0.9/ 0.15 g.L^−1^, and FH 1.5/ 0.25 g.L^−1^; **d** lignocellulosic hydrolysate 10% and 30%. Numbers account for DEGs that were differentially expressed in at least one of the two concentrations. The symbol ≠ stands for the 80 genes in which behavior changes depending on the hydrolysate concentration

The inhibitors incited a significant transcriptional response of *P. pastoris*. Out of 5040 genes found, a total of 3315 were differentially expressed (Fig. 3a). For this analysis, the threshold for statistical significance was considered an adjusted *P-*value of < 0.05 both for increasing and decreasing expression (Fig. 3a). Most genes were differentially expressed in the presence of more than one inhibitor evaluated, with the biggest differences in pattern found between furaldehydes and acetic acid than hydrolysate to the two other conditions. From the total of DEGs, 234, 66, and 959 genes were exclusively differentially expressed in the presence of acetic acid, furaldehydes, or hydrolysate, respectively. Moreover, 630 genes are common to all three inhibitors (Fig. 3a). Acetic acid induced the differential expression of 2108 (64%) genes, sharing 1228 and 16 of them exclusively with hydrolysate and furaldehydes, respectively. In the presence of furaldehydes, the yeast showed the smallest amount of DEGs, summing up to 894 (27%) genes, whereas the most amount of DEGs was found in hydrolysate conditions with 2999 (90%). Out of all DEGs found, 1194 had no annotation in the reference genome.

The yeast transcriptional response to the inhibitors was observed to be dose dependent. More specifically our results show an increase in the number of DEGs in the higher concentrations of the evaluated inhibitors (Fig. 3b–d). In the presence of acetic acid, the yeast had 2108 DEGs, with 11% and 41% of those found exclusively at either the lowest or highest concentration of the acid, respectively (Fig. 3c). Similar responses were seen for furaldehydes and hydrolysate, where 0.3% (3 genes) and 28% of DEG was exclusively for the lower concentration of inhibitor and 75% and 40% for the highest concentrations, respectively (Fig. 3b and d). However, a significant amount of common DEGs was found in both concentrations of inhibitors (Fig. 3). The number of DEGs showed that hydrolysate challenged the yeast the most, inducing the biggest change in gene expression (Fig. 3a). A total of 2999 different genes were either up or down-regulated in the presence of hydrolysate (1522 up and 1557 down-regulated) in at least one of the two concentrations employed. From 10 to 30%, a total of 80 genes changed their pattern of expression: 30 were from up- to down-regulated and 50 from down- to up-regulated, in the respective concentrations (Fig. 3d). Furaldehydes induced the smallest response in terms of the number of DEGs, summing up to 891, followed by acetic acid, with 2108 (Fig. 3).

### Central carbon metabolism

**Fig. 4:**
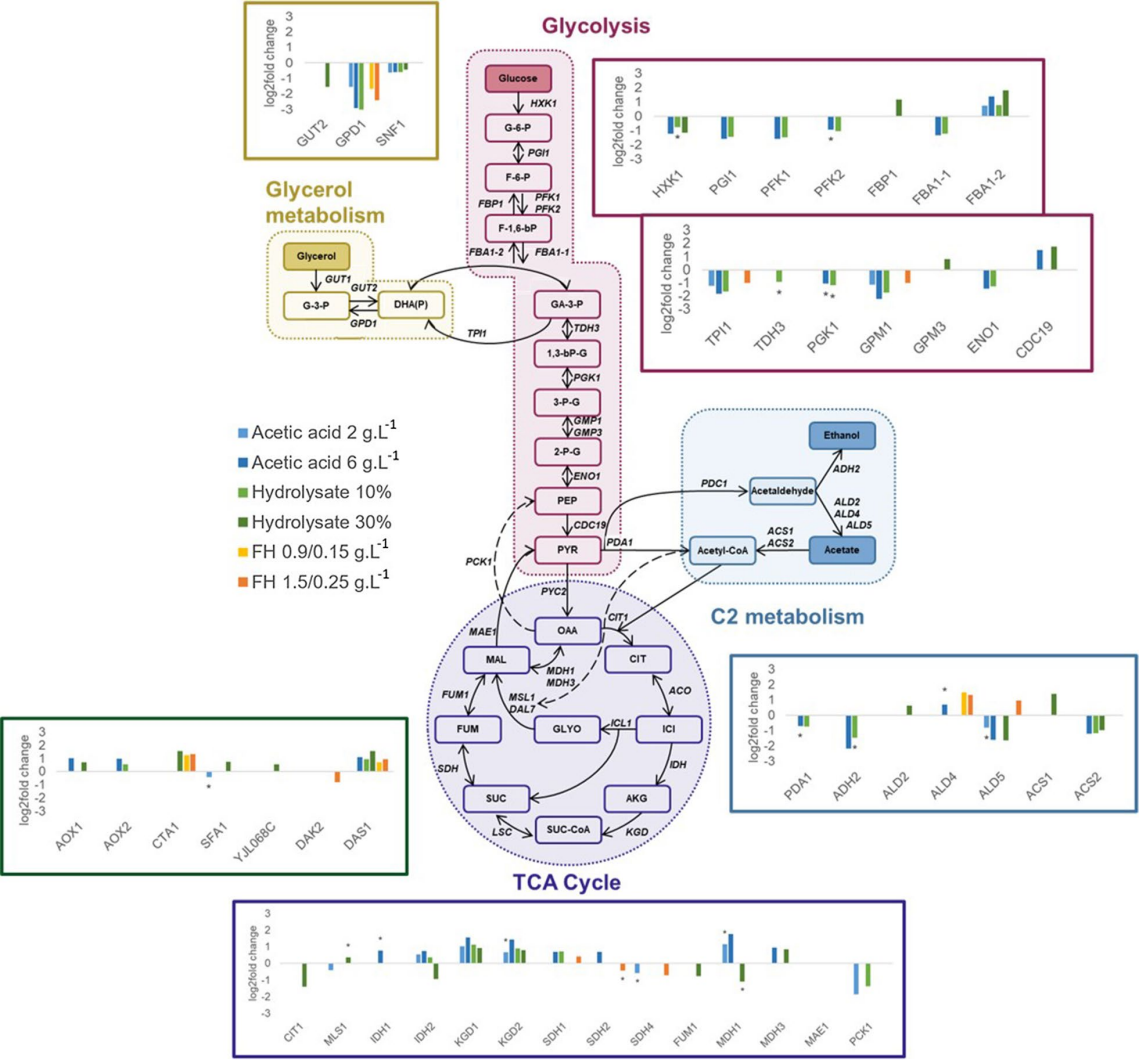
Gene expression of central carbon metabolism pathways for *P. pastoris*. Possible carbon sources are presented in colored squares: glucose; glycerol, ethanol, acetate. Bar charts represent the transcriptional changes (log2 fold) of genes in acetic acid 2 g.L^−1^ (light blue), 6 g.L^−1^ (dark blue), FH 0.9/0.15 g.L^−1^ (yellow), FH 1.5/0.25 g.L^−1^ (orange), hydrolysate 10% (light green) and 30% (dark green) with *P*-values < 0.05 or 0.1 (*on top of bar chart). Metabolites: *G-6-P* glucose 6-phosphate, *F-6-P* fructose-6-phosphate, *F-1,6-P* fructose 1,6-phosphate, *G-3-P* glycerol 3-phosphate, *GA-3-P* glyceraldehyde 3-phopshate, *1,3-bPG* 1,3-bisphosphoglycerate, *3-PG* 3-phosphoglycerate, *2-PG* 2-phosphoglycerate, *PEP* phosphoenolpyruvate, *PYR* pyruvate, *DHA(P)* dihydroxy acetone (phosphate), *OAA* oxaloacetate, *CIT* citrate, *ICI* isocitrate, *AKG* alpha-keto glutarate, *SUC* succinate, *SUC-CoA* succinyl-Coenzyme A, *FUM* fumarate, *MAL* malate, *GLYO* glyoxylate. Enzymes: *HXK1* hexokinase, *PGI1* phosphoglucose isomerase, *PFK1/2* phosphofructokinase, *FBP1* fructose-1,6-bisphosphatase, *FBA1-1/1-2* fructose 1,6-bisphosphate aldolase, *TPI1* triose phosphate isomerase, *TDH3* glyceraldehyde-3-phosphate dehydrogenase, *PGK1* 3-phosphoglycerate kinase, *GPM1/3* phosphoglycerate mutase, *ENO1* enolase I, phosphopyruvate hydratase, *CDC19* pyruvate kinase, *GUT1* glycerolkinase, *GUT2* glycerol-3-phosphate dehydrogenase, *GPD1* glycerol-3-phosphate dehydrogenase, *SNF1* central kinase, *PYC2* pyruvate carboxylase, *CIT1* citrate synthase, *ACO1/2* aconitase, *ICL1* isocitrate lyase, *DAL7* malate synthase, *IDH1/2* isocitrate dehydrogenase, *KGD1* alpha-ketoglutarate dehydrogenase complex, *KGD2* dihydrolipoyl transsuccinylase, *LSC1* succinyl-CoA ligase, *SDH1/2/4* succinate dehydrogenase, *FUM1* fumarase, *MDH1* mitochondrial malate dehydrogenase, *MDH3* malate dehydrogenase, *MAE1* mitochondrial malic enzyme, *PDC1* pyruvate decarboxylase, *PDA1* pyruvate dehydrogenase (subunit from PDH complex), *ALD2* cytoplasmic aldehyde dehydrogenase, *ALD4-1/4-2/5* mitochondrial aldehyde dehydrogenase, *ADH2* alcohol dehydrogenase, *ACS1/2* acetyl-coA synthetase, *PCK1* phosphoenolpyruvate carboxykinase. Genes or conditions with *P*-values out of the threshold were not depicted

The expression of glycolysis pathway encoding genes was overall strongly down-regulated in the presence of acetic acid and hydrolysate, with the exception of the genes *FBA1-2* (fructose 1,6-bisphosphate aldolase) and *CDC19* (pyruvate kinase) that were overexpressed (Fig. 4). Furaldehydes did not increase or reduce the expression of most glycolysis encoding genes (Fig. 4). On the other hand, genes encoding for glycerol metabolism enzymes were all inhibited, with expression levels of *GPD1* (glycerol-3-phosphate dehydrogenase) being down-regulated in presence of acetic acid, furaldehydes and hydrolysate. The C2 metabolism, i.e. acetic acid and ethanol, that integrate into the glycolysis showed a mixed pattern of expression. In general, genes encoding enzymes involved in the production of acetate and ethanol did not show differential expression or were down-regulated, such as *PDA1* (pyruvate dehydrogenase), *ADH2* (alcohol dehydrogenase), and *ALD5* (mitochondrial aldehyde dehydrogenase) (Fig. 4). Contrarily, the *ACS1* gene, which encodes an acetyl-CoA synthetase that can directly convert acetate to acetyl-CoA, and the tricarboxylic acid cycle (TCA) did not show significant differences in gene expression or were up-regulated. The most up-regulated genes from the tricarboxylic acid cycle (TCA) were found when the yeast was cultivated in presence of acetic acid, what may be related with the consumption of acetic acid seen during the cultivations (Figs. 1 and 4).

### Gene ontology analysis

**Fig. 5:**
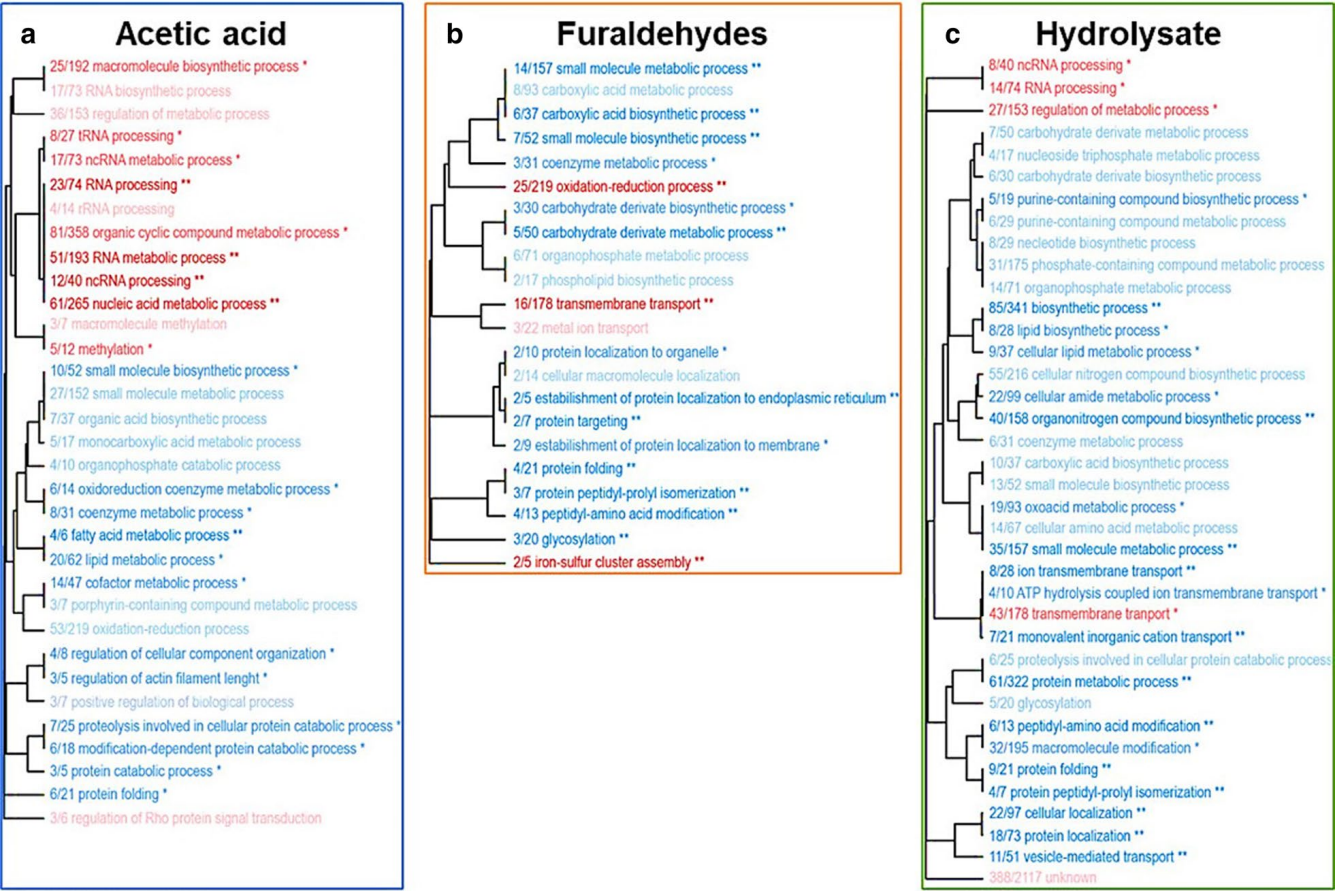
Representativeness of GO categories for the DEGs of *P. pastoris* in presence of different inhibitors. The genes with differential expression in at least one concentration of acetic acid (**a**), furaldehydes (**b**), and hydrolysate (**c**) were used in the analysis. Up and down-regulated categories are shown in red and blue, respectively. *P*-values are equal to: (**) 0.01, (*) 0.05, (no symbol) 0.1. The numbers X/Y represent the number DEGs with that GO term found in the sample by the total number of genes with that GO term in the genome

The enrichment of gene ontology (GO) categories in response to acetic acid, furaldehydes, and hydrolysate were evaluated using differentially expressed genes for each inhibitor. Acetic acid resulted in the up-regulation of DEGs in the GO categories related to nucleic acid processing, especially RNA, methylation and Rho protein signal transduction regulation (Additional file 1: Tables S1 and S2), and downregulation of oxi-reduction and macromolecules metabolic processes (Fig. 5a). From the eight genes present in the GO category methylation (*GCD10, GCD14, HSL7, MRM2, PPM1, PPM2, TGS1, PPR1*), only *GCD10* and *MRM2* were not up-regulated also in the presence of the other inhibitors evaluated (Additional file 1: Table S1). All Rho related genes were up-regulated except in the hydrolysate 30% condition (Additional file 1: Table S2).

In the presence of furaldehydes, the oxi-reduction GO category was up-regulated (Fig. 5b), as well as the GO categories transmembrane transport, metal-ion, and iron-sulfur cluster assembly (Additional file 1: Tables S3, S4 and S5). Protein-related processes, biosynthesis of small molecules (carboxylic acid), organophosphates, coenzymes, and phospholipids were expressively down-regulated (Fig. 5b). Being one of the groups found up-regulated in furaldehydes, and for its previous extensive description in literature as being relevant for tolerance to this group of inhibitors, we sought to investigate oxidation–reduction processes. We observed that DEGs found in furaldehyde conditions (FH 0.9/ 0.15 g.L^−1^ and FH 1.5/ 0.25 g.L-1) related to oxidation–reduction process and transmembrane transport GO term category were induced. Among the 5 oxidoreductases with higher increased expression (*OYE3-2, QOR2, OYE3-1, NTL100,* and *NTL101*, log twofold around 3), only *QOR2* was not flavin mononucleotide (FMN)-dependent (Table 1). Genes *ZWF1* (glucose-6-phosphate dehydrogenase) and *ALD4* (mitochondrial aldehyde dehydrogenase) reported previously as important for furaldehydes tolerance, were also found overexpressed in *P. pastoris* (Table 1).

**Table 1:** Selected differentially expressed genes (log twofold) of *P. pastoris* cultivated in presence of acetic acid, furaldehydes (HMF and furfural) and sugarcane biomass hydrolysate. In the case of *P* value > 0.05, the log2fold is not shown

Gene ID	Gene Name	Description	Acetic acid 2 g.L^−1^	Acetic acid 6 g.L^−1^	FH 0.9/ 0.15 g.L^−1^	FH 1.5/ 0.25 g.L^−1^	Hydrolysate 10%	Hydrolysate 30%	Go term associated
GQ6703442	OYE3-2*	*Conserved NADPH oxidoreductase containing flavin mononucleotide (FMN), homologous to Oye2p with different ligand binding and catalytic properties, has potential roles in oxidative stress response and programmed cell death	–	–	3.12	3.47	3.30	–	Oxidation–reduction process
GQ6700918	*PPR1*	Pyrimidine pathway regulatory protein 1	0.53	1.03	–	0.40	0.82	0.28	Macromolecule methylation, macromolecule modification, RNA metabolic process,
GQ6703443	OYE3-1*	*Conserved NADPH oxidoreductase containing flavin mononucleotide (FMN), homologous to Oye2p with different ligand binding and catalytic properties, has potential roles in oxidative stress response and programmed cell death	–	–	2.72	3.09	3.12	–	Oxidation–reduction process
GQ6701720	NTL101*	*Putative nitrilotriacetate monooxygenase family FMN-dependent oxidoreductase	–	–	2.45	2.94	2.20	–	Oxidation–reduction process
GQ6702862	*SOR1*	*Unique sorbitol dehydrogenase in *Pichia pastoris* whose promoter has activity similar to GAP promoter using different carbon sources, expression in *S. cerevisiae* is induced in the presence of sorbitol or xylose	2.26	3.77	–	1.32	3.01	2.36	Oxidation–reduction process
GQ6700554	*ALD4*	Mitochondrial aldehyde dehydrogenase; required for growth on ethanol and conversion of acetaldehyde to acetate	–	–	1.51	1.32	–	–	Oxidation–reduction process
GQ6702661	–	–	2.74	2.12	0.81	0.91	2.48	2.63	Ion transport, cation transport, transmembrane transport, metal ion transport, localization
GQ6702266	*YCT1*	High affinity cysteine transporter	–	1.35	4.43	4.81	2.87	-	Transmembrane transport, localization
GQ6703957	–	–	2.36	2.51	–	–	1.98	3.04	Transmembrane transport, localization
GQ6702465	*VBA1*	Vacuolar basic amino acid transporter 1	–	1.59	–	–	0.97	2.69	Transmembrane transport, localization
GQ6702095	*TPO3*	Polyamine transporter 3	2.53	1.81	–	–	1.98	2.63	Transmembrane transport, localization
GQ6703034	–	–	–	2.01	1.22	1.32	1.20	2.56	Transmembrane transport, localization
GQ6703337	*DUR3-2	*Plasma membrane transporter for both urea and polyamines, expression in *S. cerevisiae* is highly sensitive to nitrogen catabolite repression and induced by allophanate, the last intermediate of the allantoin degradative pathway	–	2.34	1.32	4.92	–	1.14	Transmembrane transport, localization, localization,
GQ6705065	–	*Hypothetical protein not conserved	4.83	3.77	4.66	4.50	1.94	2.34	–
GQ6702974	–	*Hypothetical protein not conserved	2.81	2.71	2.90	3.90	-	1.54	–
GQ6700086	*RPH1	*JmjC domain-containing histone demethylase	2.80	2.85	2.84	2.43	0.97	1.15	–
GQ6701722	*QOR2	*Putative quinone oxidoreductase (NADPH:quinone reductase), similar to *Scheffersomyces stipitis* QOR2, and similarity to Zinc-binding dehydrogenases	–	–	2.86	–	2.73	3.22	Oxidation–reduction process,
GQ6701721	*NTL100	*Putative nitrilotriacetate monooxygenase family FMN-dependent oxidoreductase	–	–	2.52	–	2.69	2.96	Oxidation–reduction process
GQ6701716	*CAO1	*Copper amine oxidase similar to *Schizosaccharomyces pombe* CAO1	–	2.90	-	6.17	–	–	Amine metabolic process, oxidation–reduction process
GQ6702273	*DAL1	*Allantoinase, expression in *S. cerevisiae* sensitive to nitrogen catabolite repression	2.36	3.73	2.59	2.78	–	–	Oxidation–reduction process
GQ6705251	*-*	*Hypothetical protein conserved (domain: GAL4)	2.75	3.12	2.81	2.56	–	–	Regulation of metabolic process, biological regulation
GQ6701500	*SOA1-6	*Putative protein with similarity to allantoate permease, similar to the allantoate permease (Dal5p) subfamily of the major facilitator superfamily	–	–	2.18	–	2.51	3.08	Transmembrane transport, localization, localization,
GQ6703338	*AMD2*	–	–	2.65	1.89	4.47	–	1.66	–
GQ6701041	*CAR1*	–	–	2.36	-	3.85	–	1.89	–
GQ6703874	*NCS6*	–	2.79	2.79	2.80	1.53	–	1.54	–
GQ6704853	*PIC2*	Mitochondrial phosphate carrier protein 2 (Phosphate transport protein 2) (PTP 2) (Pi carrier isoform 2) (mPic 2)	2.75	3.38	2.88	2.67	–	-	–
GQ6702197	*PST2*	Protoplast secreted protein 2	-	-	4.56	1.27	–	0.90	–
GQ6703286	*STP3*	Zinc finger protein STP3	4.84	3.91	4.40	3.90	–	-	–
GQ6700345	*ZWF1*	Glucose-6-phosphate dehydrogenase (G6PD)			0.74	0.67			Single-organism carbohydrate metabolic process, small molecule metabolic process, oxidation–reduction process, hexose metabolic process,

*information manually annotated

Most identified GO terms found for lignocellulosic hydrolysate were down-regulated, being RNA processing, regulation of metabolic processes, and transmembrane transport the only up-regulated-related terms (Fig. 5c). A total of 25 DEGs within the transmembrane transport GO group were up-regulated in both hydrolysate conditions (Additional file 1: Table S6), with 6 genes at least five times up-regulated in at least one of the two concentrations. (Table 1).

**Fig. 6:**
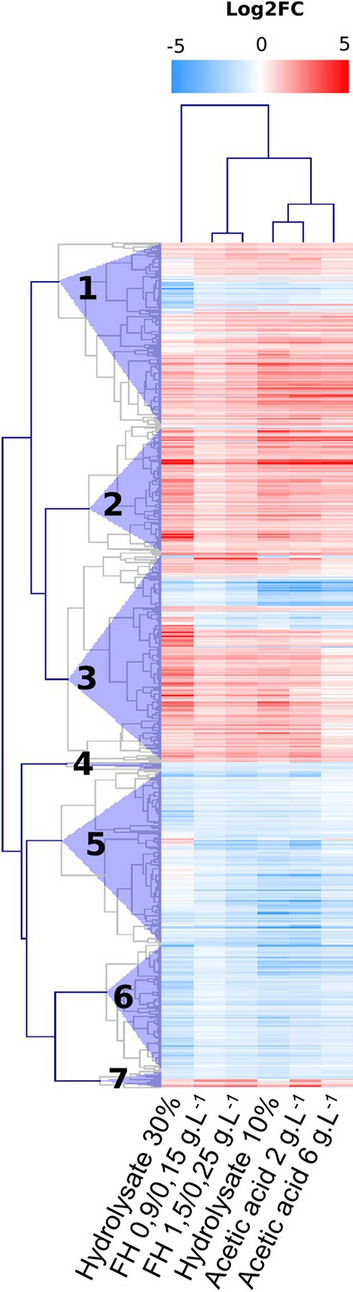
Hierarchical clustering heat map of the 630 DEGs common in all categories. Changes in the expression are shown on a color scale, where red represents up-regulation and blue represents down-regulation. Each column relates to one inhibitory condition and each row represents one DEG

To get a better insight of the yeast typical response to the different inhibitors, a heat map was constructed with the 630 DEGs found for all three inhibitors (Fig. 3a) in at least one of the two concentrations evaluated (Fig. 6). The gene expression profiles from the cells grown in the presence of 2 g.L^−1^ and 6 g.L^−1^ acetic acid and hydrolysate 10% are more like to each other than to cells grown in hydrolysate 30%. This difference is consistent with the physiological (Fig. 1) and PCA analysis results (Fig. 2). Additionally, seven distinct clusters of differentially expressed genes and enriched GO annotations are found in the heat map (Fig. 6). Cluster 2 and most of 7 involve up-regulated categories mainly related to regulation of transcription from RNA pol II promoter, intracellular signal transduction, and nucleobase-containing compound metabolism. Most genes on cluster 7 were up-regulated, but a small part, especially in presence of acetic acid 6 g.L^−1^ was down-regulated. Cluster 4, 5, and 6 include down-regulated GO categories (except hydrolysate 30%) mostly related to transport, especially vesicle-mediated transport, regulation, initiation of transcription, and tRNA aminoacylation for protein translation. Clusters 1 and 3 showed both up and down-regulated categories, those related to nucleotides biosynthesis and oxidation–reduction processes.

The 10 genes most up-regulated in each condition, i.e. acetic acid, furaldehydes, and hydrolysate, in the two different concentrations, were identified (Additional file 1: Table S7). From those 60 genes, 26 were present in more than one condition, usually in the lower and higher concentration of the same inhibitor. Thus, a total of 34 unique genes were identified. Among those genes, 19 (*DUR3-2, GQ6705065, GQ6702974, RPH1, QOR2, OYE3-2, NTL100, CAO1, DAL1, GQ6705251, SOA1-6, AMD2, CAR1, NCS6, PIC2, PST2, SOR1, STP3,* and *YCT1*) were up-regulated in all tested conditions (Table 1).

## Discussion

The *P. pastoris* performance in the presence of acetic acid, furaldehydes, and sugarcane hydrolysate shown here for the first time demonstrates its relatively high tolerance to lignocellulose-derived inhibitors, especially to acetic acid. Higher concentrations of furaldehydes (0.25 g.L^−1^ HMF, 1.5 g.L^−1^ furfural) or acetic acid 6 g.L^−1^ hampered but did not impair *P. pastoris* growth. The total inhibition of yeast metabolism was only observed in the presence hydrolysate 30%, which contains 6 g.L^−1^ of acetic acid and FH 1.5/ 0.25 g.L^−1^ besides other compounds. These synergistic effects of lignocellulose-derived inhibitors have also been shown for *S. cerevisiae* and other yeasts (Modig et al. 2002; Liu et al. 2004; Almeida et al. 2007; Skerker et al. 2013). Yeast tolerance to the inhibitors has been shown to be species and strain-specific (Modig et al. 2008; Sitepu et al. 2015). While some *S. cerevisiae* strains have shown sensibility to as few as 1 g.L^−1^ of furaldehydes (Almeida et al. 2007) and 4,8 g.L^−1^ acetic acid (Ludovico et al. 2001), others have shown to be tolerant to concentrations as high as 10 g.L^−1^ (Stratford et al. 2013). *O. polymorpha,* a methylotrophic yeast, showed the capacity to grow and produce xylitol in wheat straw hydrolysate containing up to 12.24 g.L^−1^ of acetic acid and 4.17 g.L^−1^ of total phenolics (Yamakawa et al. 2020). Although direct comparison of the yeasts is not possible due to the diversity of experimental conditions employed, the results reported here demonstrated that *P. pastoris* can withstand lignocellulose-derived inhibitors even when inoculated at low cell density (DO_600_ 5).

*P. pastoris* showed a dose-dependent response to acetic acid, furaldehydes, and hydrolysate at the physiological and transcriptional levels. Increased concentrations of acetic acid, furaldehydes, and hydrolysate lead to stronger inhibitory effects on yeast metabolism, increasing the time for the yeast to complete sugar consumption and grow. A similar response has been shown for *S. cerevisiae* (Pampulha and Loureiro-Dias 1990; Larsson et al. 1999; Palmqvist et al. 1999; Liu et al. 2005; Dong et al. 2017)*.* The conversion of furaldehydes took place within few hours of cultivation. When the inhibitory effects were absent, the yeast exited lag phase, and started to consume sugars and grow (Fig. 1). For all conditions, a positive correlation was found between the increased concentration of the inhibitor, the physiological impairment of the yeast’s growth and the number of DEGs (Figs. 1 and 3). These results are supported by similar observations for *S. cerevisiae* (Dong et al. 2017; Li et al. 2020).

*P. pastoris* responds to lignocellulose-derived inhibitors by increasing oxidative stress response. Genes related to methylation up-regulated in presence of acetic acid were also up-regulated in other conditions but to a lesser extent (Additional file 1: Table S1). Overexpression of methyltransferases such as *PPR1* has been shown to improve *S. cerevisiae* growth and fermentation performance in the presence of acetic acid, presumably due to the reduced intracellular accumulation of reactive oxygen species (Zhang et al. 2015). Since reactive oxygen species are also generated in the presence of furaldehydes (Gorsich et al. 2006), *PPR1* up-regulation in the presence of such compounds may also be advantageous. Interestingly, in this work, *PPR1* was up-regulated in all conditions evaluated, doubling its expression in acetic acid 6 g.L^−1^. Another up-regulated group was the regulation Rho protein signal transduction. Rho is a family of proteins which regulation affects numerous cell processes (Etienne-Manneville and Hall 2002) and is essential for osmotic stress response (Annan et al. 2008) and low pH survival in yeast (Fletcher et al. 2015). However, further evaluation of the Rho role in lignocellulose-derived inhibitor tolerance must be performed.

The increased expression of oxidoreductases seen when the yeast was cultivated in the presence of furaldehydes (Fig. 5) might be associated with the conversion of HMF and furfural to their less toxic forms, as reported previously for other yeasts (Horváth et al. 2001; Liu et al. 2004; Petersson et al. 2006; Almeida et al. 2008). Among relevant oxidoreductase encoding genes found up-regulated in this work (Table 1), *ZWF1* (Gorsich et al. 2006) and *ALD4* (Liu 2011) have been reported previously as capable of reducing HMF and furfural toxicity to the cell (Heer et al. 2009; Ma and Liu 2010). Other oxidoreductases potentially involved in the detoxification of furaldehydes, but previously not shown, are OYE3, QOR2 and NTL100 (Table 1). Another gene possibly related to furaldehydes tolerance is *YCT1* (Table 1), which encodes a cysteine transporter found to be up-regulated in the presence of many inhibitors in the yeast *Kluyveromyces marxianus* (Wang et al. 2018). Cysteine is related to the synthesis of glutathione, which is an important antioxidant molecule related to detoxification and oxidative stress response to HMF and furfural (Fauchon et al. 2002; Ask et al. 2013). In fact, glutathione importance in detoxification has also been related to the synthesis of sulfur amino acids and saving mechanisms in yeast (Fauchon et al. 2002), which may explain the iron-sulfur GO category up-regulation in presence of furaldehydes. Thus, *ZWF1, ALD4, OYE3, QOR2, NTL100 YCT1,* and *PPR1* are potential candidates for improving *P. pastoris* tolerance to the lignocellulose-derived inhibitors.

*P. pastoris* was able to co-consume glucose and acetic acid (Fig. 1b), which is not observed in most strains of *S. cerevisiae* (Sousa et al. 2011). This could be correlated with the reduced glucose consumption rate and toxicity of acetate (Fig. 1b). Acetic acid may lead to the cytosol acidification by its dissociation in the cytosol, affecting cell metabolism and survival (Pampulha and Loureiro-Dias 1989; Sousa et al. 2011; Rego et al. 2014). The overexpression of genes responsible for the consumption and conversion of acetic acid may be a strategy to reduce its toxicity. This is corroborated by the transcriptional data that demonstrated that *P. pastoris* repressed glycolytic pathway and up-regulated C2/C3 metabolism in presence of inhibitors, especially in the presence of acetic acid and hydrolysate (Fig. 4). These results are further supported by the recent results of Xu and coworkers (Xu et al. 2019) who had recently demonstrated that *P. pastoris* is capable to metabolize acetate in presence of glucose. The experimental data published by the authors does not explicitly demonstrate the co-consumption of glucose and acetate (as reported in here); however, metabolite analyses suggest so. In addition, contrary to *P. pastoris*, *S. cerevisiae* shows a Crabtree effect positive metabolism i.e. presents a fermentative metabolism even when it is cultivated in aerobiosis when glucose is present in high concentrations in the medium (Crabtree effect). Thus, *S. cerevisiae* show a diauxic shift, where it switches from rapid fermentative growth once the preferred carbon source (glucose) has been exhausted to slower exponential growth by aerobic respiration using ethanol/acetate as carbon sources.

In conclusion, our study presents the first physiological and genome-wide transcriptome analysis of *P. pastoris* under the effect of major inhibitors found in the lignocellulosic hydrolysate. The results reveal that acetic acid, furaldehydes, and sugarcane hydrolysate inhibit the cell metabolism in a dose-dependent manner, and the yeast transcriptional response increases with the increased concentrations of the inhibitors. Acetic acid can be co-consumed by the yeast as an alternative carbon source, although it affects yeast’s growth. Even though *P. pastoris* is a well-known and one of the favorite host organisms used as a tool in both academia and industry, little is known about its response to toxic compounds, and especially those present in lignocellulosic hydrolysate. Therefore, the results reported here are useful to expand the use of cheap carbon sources (like lignocellulosic hydrolysate) in bioprocesses employing this yeast. Gene clusters related to the response of *P. pastoris* to lignocellulose-derived inhibitors are described here for the first time, and candidate genes to improve yeast tolerance were identified.

## Supplementary Information

**Additional file 1:** **Table S1.** DEGs found in acetic acid containing conditions related to methylation processes (GO-Biological Process). **Table S2.** DEGs found in acetic acid containing conditions related to regulation of Rho protein signal transduction (GO-Biological Process). **Table S3.** DEGs found in furaldehydes containing conditions related to transmembrane transpor transport (GO-Biological Process). **Table S4.** DEGs found in furaldehydes containing conditions related to metal-ion transport (GO-Biological Process). **Table S5.** DEGs found in furaldehydes containing conditions related to iron-sulfur cluster assembly (GO-Biological Process). **Table S6.** DEGs found in hydrolysate containing conditions related to transmembrane transport (GO-Biological Process). **Table S7.** Compilation of top 10 most overexpressed genes in each of the evaluated conditions (GO-Biological Process).

## Data Availability

The transcriptome datasets generated during the current study are available in the NCBI with the accession number PRJNA666642.
